# Preventing Multidrug-Resistant Bacterial Transmission in the Intensive Care Unit with a Comprehensive Approach: A Policymaking Manual

**DOI:** 10.3390/antibiotics12081255

**Published:** 2023-07-30

**Authors:** Georgios Schinas, Elena Polyzou, Nikolaos Spernovasilis, Charalambos Gogos, George Dimopoulos, Karolina Akinosoglou

**Affiliations:** 1 Department of Medicine, University of Patras, 26504 Patras, Greece; georg.schinas@gmail.com (G.S.); polyzou.el@gmail.com (E.P.); cgogos@med.upatras.gr (C.G.); akin@upatras.gr (K.A.); 2 Department of Internal Medicine and Infectious Diseases, University General Hospital of Patras, 26504 Patras, Greece; 3 Department of Infectious Diseases, German Oncology Center, 4108 Limassol, Cyprus; 4 3rd Department of Critical Care, Evgenidio Hospital, Medical School, National and Kapodistrian University of Athens, 11528 Athens, Greece; gdimop@med.uoa.gr

**Keywords:** intensive care unit, multidrug-resistant bacteria, transmission, colonization, healthcare personnel

## Abstract

Patients referred to intensive care units (ICU) commonly contract infections caused by multidrug-resistant (MDR) bacteria, which are typically linked to complications and high mortality. There are numerous independent factors that are associated with the transmission of these pathogens in the ICU. Preventive multilevel measures that target these factors are of great importance in order to break the chain of transmission. In this review, we aim to provide essential guidance for the development of robust prevention strategies, ultimately ensuring the safety and well-being of patients and healthcare workers in the ICU. We discuss the role of ICU personnel in cross-contamination, existing preventative measures, novel technologies, and strategies employed, along with antimicrobial surveillance and stewardship (AMSS) programs, to construct effective and thoroughly described policy recommendations. By adopting a multifaceted approach that combines targeted interventions with broader preventive strategies, healthcare facilities can create a more coherent line of defense against the spread of MDR pathogens. These recommendations are evidence-based, practical, and aligned with the needs and realities of the ICU setting. In conclusion, this comprehensive review offers a blueprint for mitigating the risk of MDR bacterial transmission in the ICU, advocating for an evidence-based, multifaceted approach.

## 1. Introduction

The invisible threat of multidrug-resistant (MDR) bacterial cross-contamination among intensive care unit (ICU) personnel poses a significant challenge for healthcare facilities. Key insights from research on healthcare-acquired colonization (HAC) and infection (HAI) underscore the prevalence of MDR isolates, particularly among patients requiring mechanical ventilation [1]. Extensive research has been conducted to understand the dynamics of MDR bacterial cross-contamination in the ICU setting. Studies have identified various independent factors that contribute to the transmission of these pathogens, including inadequate hand hygiene, improper use of personal protective equipment, and contaminated medical devices [2,3,4].

The occurrence of healthcare personnel (HCP) colonization, often facilitated by inadequate hand hygiene, the improper use of personal protective equipment, and contaminated medical devices, can have significant implications for patient care. Moreover, the role of healthcare workers in cross-contamination chains, with mobile phones identified as potential vectors [5], highlights the complex dynamics of bacterial transmission within the ICU [6,7,8,9]. Studies have indicated that inanimate surfaces and equipment, such as bedrails, stethoscopes, and medical charts, can become contaminated with MDR bacteria in ICUs. This, in turn, potentially contributes to the transmission of pathogens to patients, primarily via the hands of the HCP [10].

Preventive measures such as isolation protocols and environmental disinfection techniques are pivotal for reducing MDR bacterial cross-contamination and subsequent transmission. However, challenges in maintaining compliance have consistently been reported. Therefore, monitoring adherence to hand hygiene and providing timely feedback are crucial components of every hygiene protocol in the ICU. Novel technologies, including advanced disinfection methods and stringent monitoring techniques, can further reduce the impact of HCP on MDR bacterial transmission [11,12]. Furthermore, advancements in healthcare design and hospital engineering have shown promising potential for tackling the spread of MDR within the ICU [13].

The importance of antimicrobial surveillance and stewardship (AMSS) along with preventive measures is critical. Evidence suggests that a systematic and vigilant approach to monitoring antimicrobial use could have significant benefits, especially among ICU patients [14]. Healthcare professionals should embrace them as an integral part of their practice to limit the emergence, colonization, and subsequent transmission of MDR bacteria. Current policy recommendations emphasize the necessity for continued improvements in hand hygiene compliance, leveraging innovative technologies to overcome obstacles in preventing transmission and assessing adherence to protocols [13]. Incorporating the monitoring and assessment of hygienic-targeted practices as a principal component of management strategies is paramount in the complex ICU environment. Adjusting practices based on data-driven insights and evidence-based recommendations to reduce the risk of MDR contraction ensures the efficacy of interventions and the accuracy of policy measures.

This review aims to provide a comprehensive overview of the current knowledge in understanding the cross-contamination patterns of MDRs in the ICU and provide the blueprint for establishing efficient policy recommendations to tackle them. We examined the underlying mechanisms through which personnel colonization contributes to the dissemination of MDR bacteria within the ICU setting and proposed effective measures to mitigate the associated risks and broader implications for patient care. By establishing a clear understanding of the intricacies involved in bacterial transmission, adherence monitoring, and quality surveillance, we aimed to provide essential guidance for the development of robust prevention strategies, ultimately ensuring the safety and well-being of patients and healthcare workers in the ICU setting.

## 2. The Path of the ICU-Acquired Infections

ICU-acquired infections, particularly those involving MDR bacteria, represent a major challenge for healthcare systems worldwide [15]. Healthcare workers’ hands often serve as the primary vectors for cross-transmission, connecting contaminated surfaces and equipment to patients, which may result in colonization or infection [7,10]. Other factors that play a role in contamination and cross-transmission rates in the ICU may include nurse-staffing levels, frequency/number of colonized or infected patients, ICU structural features (e.g., single-bed or multi-bed ICU rooms), and the adoption of antimicrobial stewardship programs (ASPs) [16,17]. However, approximately 20–40% of nosocomial infections are estimated to arise from cross-transmission via healthcare workers’ hands [7,10]. Moreover, the hands of caregivers tend to exhibit greater contamination over time, marking progression from sufficient care duration [18].

In a recent systematic review, HCP hands were found to be frequently contaminated with MDR organisms across all care settings, with a prevalence ranging from 4% to 9% [19]. The researchers observed a slightly higher rate of *Pseudomonas aeruginosa* (5.67%) and *Acinetobacter baumannii* (7.52%) in ICU settings than in inpatient floors (3.93% and 5.60%, respectively), but a lower rate of MRSA in the ICU (2.20%) than in the inpatient group (5.14%). The highest rates of MRSA were found in North America (8.28%), followed by Asia (4.23%) and Europe (2.47%). At first glance, the reported rates may seem low; however, it is important to consider the influence of study design and measurement on MDR prevalence rates. Cross-sectional studies, for example, showed a pooled prevalence of 3.25% compared with 12.49% for other designs, such as randomized controlled studies and pre-post studies. The method of culturing also seems to affect the observed prevalence rates. The juice glove and swab methods had consistently higher observed rates, ranging from 4.48% to 6.90%, whereas the agar direct contact method had the lowest prevalence rate (1.55%) [19].

The delineation of a marked patient zone is a well-known concept that has been shown to improve hand hygiene compliance among healthcare personnel and can help understand cross-contamination patterns within the vibrant ICU environment [20]. The contamination of surfaces within the patient zone, an area that encompasses the patient and their immediate surroundings, can arise either via the contaminated hands of healthcare workers or the direct patient shedding of microorganisms [21]. Higher environmental contamination has been reported around infected patients than around patients who are only colonized, with a correlation between the frequency of environmental contamination and culture-positive body sites [17,22]. It has also been shown that MDR bacteria can contaminate communal surfaces, the surfaces of medical equipment, and other high-contact zones in ICUs. In a randomized crossover study, the recontamination of high-contact surfaces in ICUs occurred 4 h after standard cleaning measures [23]. Various ICU equipment items and commonly used and/or touched objects harbor bacteria, often showing antibiotic susceptibility profiles akin to those found among patients. For instance, electrocardiography lead wires have demonstrated contamination rates between 20% and 45% [24,25]. Stethoscopes are heavily contaminated, with bedside ones at 95% and personal ones at 67% [26]. Ventilator surfaces are almost entirely polluted with bacterial contamination ranging from 70.6% to 100% [27]. Portable radiography and ultrasound equipment, despite cleaning, show varied and sometimes residual contamination [8,28,29,30]. Computer keyboards and faucet handles in the ICU setting have also been found to be significant reservoirs of nosocomial pathogens, with the colonization rate for keyboards reaching 26% in occupied rooms and faucet handles exhibiting rates of 15% [31]. Medical charts are also hotspots for bacteria, with contamination rates ranging from 80% to 90% [32,33,34]. Lastly, even personal items, such as mobile phones, present a high risk, boasting contamination rates of up to 94.5% [35].

The survival of MDR bacteria on surfaces varies significantly among species, ranging from hours to months or even years, depending on the species, as does their ability to persist with both environmental input and various cleaning methods. According to the latest comprehensive systematic review of the persistence of bacteria on inanimate surfaces, Kramer et al. reported that resilient *Acinetobacter* spp., notorious for their environmental tenacity, can maintain their presence on surfaces for durations ranging from three days to five months [36]. The ubiquitous *Escherichia coli* presents a survival period that is as variable as it is long, persisting anywhere from 1.5 h to an extensive 16 months. *Klebsiella* spp., another prominent member of the *Enterobacteriaceae* family, outpaces *E. coli*, enduring from 2 h to 30 months. In Gram-positive bacteria, *Enterococcus* spp., which include both vancomycin-resistant and vancomycin-sensitive strains (VRE and VSE), can persist for 5 days to 4 months. *Staphylococcus aureus*, encompassing its methicillin-resistant variant (MRSA), exhibits survival periods ranging from a week to a robust seven months [36]. This wide range of durability and persistence on surfaces among MDR pathogens underscores the importance of meticulous hygiene practices in healthcare settings, as well as the necessity for comprehensive sterilization protocols in high-risk environments such as ICUs.

## 3. Hand-Hygiene

The essential practices for preventing HAIs in the ICU have been comprehensively addressed by the recently published update of “Strategies to Prevent Healthcare-associated Infections through Hand Hygiene” by the Society for Healthcare Epidemiology of America (SHEA), which was developed through a robust collaborative effort by numerous prominent organizations [37]. Practical recommendations to ensure hand hygiene in acute-care settings include promoting healthy hand skin and fingernails, using alcohol-based hand sanitizers (ABHS) in most clinical situations, performing hand hygiene as indicated by the CDC or WHO Five Moments (before touching a patient, before cleaning/aseptic procedures, after body fluid exposure/risk, after touching a patient, and after touching patient surroundings) [38], maintaining short and natural fingernails, and providing healthcare personnel with accessible hand moisturizers for the primary and secondary prevention of dermatitis. Other essential practices include selecting appropriate hand hygiene products (liquid, gel, or foam ABHS with at least 60% alcohol for routine hand hygiene), ensuring the accessibility of hand hygiene supplies, appropriate glove use (proper gloving technique, easy doffing techniques, and hand hygiene after glove removal), and taking steps to reduce environmental contamination associated with sinks and sink drains. Studies have shown that modifications in sink design, particularly increasing the depth of the sink bowl and reducing high water flow rates, can significantly reduce the risk of pathogen dissemination [39]. Another important aspect of sink hygiene is to dedicate a number of sinks exclusively to hand washing. However, owing to logistical complexities, a more practical approach would be to refrain from disposing of substances that promote the growth of biofilms (e.g., intravenous solutions, medications, food, or human waste) in handwashing sinks.

Maintaining adherence to hand hygiene is of paramount concern in the multimodal environment of ICUs. A recent study and meta-analysis from China evaluated the overall hand hygiene compliance (HHC) rate and characteristics during the COVID-19 pandemic and compared them with those before the outbreak [40]. The study concluded that during the COVID-19 pandemic, HHC showed a significant improvement, following widespread interventions to improve hand hygiene compliance among healthcare providers, including strengthening education, increasing monitoring frequency, providing feedback, and using automatic monitoring systems. HHC was found to be the highest among nurses (80%), followed by doctors (76%) and auxiliary workers (70%). The study also identified that HHC was highest after contact with the body fluids of patients (91%) and lowest before contact (68%) [40].

Monitoring techniques that could potentially evaluate such adherence may include direct overt and covert observations, automated hand hygiene monitoring systems (AHHMSs), and remote video observation. Although each method has its strengths and weaknesses, it is posited that automated systems hold immense promise for ICUs. Direct observation, either overt or covert, is fraught with potential biases and limitations. Remote video observations are encumbered by privacy concerns and logistical issues. Hence, electronic event counters or wireless connections with dispensers and badges, such as AHHMSs, are effective solutions in ICU settings.

A recent study aimed to describe the hand hygiene activities of healthcare workers in the rooms of ICU and non-ICU patients using an automated monitoring system before and after the onset of the COVID-19 pandemic [41]. The system employed allowed for the continuous evaluation of hand hygiene compliance without the need for direct observation, reducing the burden on staff and ensuring accurate data collection, as highlighted by its findings. The study observed a significant increase in alcohol-based hand sanitizer (ABHS) consumption in the Department of Medicine during the COVID-19 pandemic compared to the baseline period. However, the ABHS consumption in the ICU remained relatively constant throughout the study period. Moreover, ABHS consumption was higher in the rooms of COVID-19 patients in both the Department of Medicine and ICU during the pandemic waves, and multivariate analysis showed that ABHS consumption was associated with the number of HCP in the ICU. All findings were consistent with hand hygiene practices in different hospital departments, and the results accurately reflected the effectiveness of ongoing training programs and coaching by infection control staff, underscoring the accuracy of AHHMS in collecting real-time ABHS consumption data [41].

Another study by Hess et al. examined the impact of the COVID-19 pandemic on the use of an electronic hand hygiene monitoring system (EHHMS) and HHC in a hospital setting [42]. The researchers found that the use of the EHHMS decreased during the pandemic, largely due to shortages of hand hygiene products and changes in personal protective equipment procedures. Despite this, overall HHC increased during the pandemic, particularly in COVID-19 patient units. The study also found that the use of badges, which record individual hand hygiene events, decreased by 44% after the pandemic. The researchers concluded that, while hand hygiene is recognized as important, barriers to badge use may prevent participation in the EHHMS [42].

Overall, AHHMSs leverage technology, allowing for the continuous monitoring and capture of an extensive number of hand hygiene opportunities, despite potential limitations in perceived accuracy, staff acceptance, and cost implementation [43]. These systems can potentially revolutionize adherence measures, aligning them with the fast-paced, high-demand environment of ICUs [44].

## 4. Environmental Cleaning

In the ICU setting, various interventions have been employed to ensure proper and efficient environmental cleaning to reduce the cross-contamination of personnel with MDRs. Overall, general recommendations for environmental cleaning and disinfection practices in ICUs are summarized in Table 1 based on a recently published report from the Koch Institute [45] and in Table 2 based on a recently published report from a committee that was constituted by the Ministry of Health and Family Welfare of the government of India [46] to better reflect the situation in a developing country.

Different environmental hygiene interventions can be classified into three major categories: mechanical, chemical, and human factors. Mechanical interventions such as protective isolation using plastic isolators and air curtains, negative pressure ventilation in patient rooms, ultraviolet (UV) disinfection, and portable high-efficiency particulate absorption (HEPA) filters have shown some success in reducing specific MDR infections and minimizing bacterial presence on various surfaces and equipment in particular settings [47]. For example, protective isolation in a burn unit using plastic isolators and air curtains demonstrated a lower incidence of *Pseudomonas aeruginosa* infection than in open wards [48]. However, other microorganisms appeared at similar rates in the isolators and in the open wards [48]. Another study evaluated the effectiveness of an enhanced cleaning protocol involving the use of UV-C irradiation in a hospital ICU and high-risk areas. The study found that the protocol significantly reduced the presence of microbial contaminants on ICU surfaces from 64.3% to 17.5% [49]. Finally, the installation of HEPA filters led to a reduction in HAI rates and invasive fungal infections in the hematology unit [50]. Interestingly, this is the only study we were able to identify that calculated the cost-effectiveness of such an intervention, with the median cost per patient in U.S. dollars significantly decreasing following the installation of the filters [50].

Chemical interventions targeting the environmental reservoirs of MDRs using chemical cleaning agents comprise the backbone of sterilization efforts. A variety of active substances and formulations have been tested over the years, including ethanol, propanol, formaldehyde, peroxides, inorganic chlorine releasers, and phenol derivatives. When selecting disinfectants for use in ICUs, it is important to consider their effectiveness against various microorganisms, including bacteria, viruses, yeasts, mold spores, and bacterial spores [51]. For instance, products containing ethanol (>80% *w*/*w* or in synergistic combination) are highly effective against both Gram-positive and Gram-negative bacteria, enveloped and partially lipophilic viruses, and yeasts. However, they have little to no effect on mold spores or hydrophilic viruses. Formaldehyde-based formulations, however, are highly effective against all types of microorganisms listed, including bacterial spores, viruses, yeasts, and mold spores. Nonetheless, the effectiveness of these substances depends not only on the active ingredient but also on the formulation and exposure time.

Several novel technologies and strategies have emerged with the potential to improve cleaning and decontamination efforts using both mechanical and chemical approaches. Aerosolized agents, such as hydrogen peroxide dry-mist and vapor, have recently gained ground by demonstrating promising results in eradicating pathogens, such as *Clostridioides difficile*, MRSA, VRE, *A. baumannii*, *Serratia*, mycobacteria, and viruses [52]. Moreover, antimicrobial surfaces coated with copper or silver can be applied to high-touch areas to reduce the risk of cross-transmission, and novel automated ultraviolet radiation devices have shown effectiveness in reducing contamination on commonly touched hospital surfaces [53]. ICU rooms with copper alloy-coated objects showed lower rates of HAI and colonization by MDR bacteria [54,55]. Utilizing these innovations to complement manual cleaning methods, especially in readily contaminated areas or during outbreaks, may prove beneficial in improving hygiene and reducing risks. Despite these advances, further research is needed to determine the true role of these technologies in environmental hygiene, considering their logistical complexity and associated costs.

## 5. Identification of Risk Factors for Colonization

Considering the different effectiveness of prevention strategies for specific bacterial species, further exploration is needed to determine the most effective measures to prevent the acquisition and spread of MDR bacterial colonization. High colonization pressure is often associated with the spread of multidrug-resistant organisms in healthcare settings, as it reflects the increased potential for the cross-transmission of these organisms among patients. In a single-center prospective cohort study, colonization pressure was identified as an independent risk factor for MDR bacteria in the ICU (OR (95% CI) 4.18 (1.03–17.01), *p* = 0.046) [56]. Other independent risk factors were mechanical ventilation (3.08 (1.28–7.38), *p* = 0.012) and arterial catheter use (OR, 3.04 (1.38–6.68); *p* = 0.006) [56]. However, the marginal significance of the finding, along with the author’s failure to adjust for multiple comparisons despite the multivariate analysis design, raises questions about the robustness of the reported results.

The identification of patient risk factors for MDR bacterial colonization in ICUs is a proposed measure that may serve both as an effective prevention intervention and a treatment strategy in specific patient populations, such as immunocompromised patients. However, a prospective study investigating the accuracy of the American Thoracic Society (ATS)/Infectious Diseases Society of America (IDSA) criteria in predicting infection or colonization by MDR organisms in ICU patients revealed that the criteria demonstrated high sensitivity and negative predictive values but low specificity and positive predictive values in predicting MDR colonization or infection at ICU admission [57]. According to these findings, independent predictors of infection or colonization with MDR bacteria upon ICU admission included prior antimicrobial treatment, residence in a nursing home, and prior hospitalization. Another study from China demonstrated the low accuracy of ATS guidelines in predicting MDR colonization, particularly in patients from departments with low or high MDR infection rates, further challenging their role as independent interventions and questioning their clinical value in ICU settings [58].

Overall, screening techniques have inherent limitations. For instance, they may not detect all carriers of MDR bacteria, especially if the bacteria are present in low numbers or at sites not sampled. Additionally, false negatives can occur because of limitations in the sensitivity of screening tests, and the costs associated with multiple and repeated tests cannot be undermined. Therefore, while screening is a crucial component of infection control, it should be complemented with other strategies, such as identifying risk factors for colonization and spread in ICU patients upon admission, for improved management and infection control.

## 6. ICU Design Workflow

Adapting the ICU infrastructure and functional processes has also been proposed as a measure to combat potential outbreaks and reduce cross-contamination. The comprehensive reassessment and potential restructuring of the ICU’s architecture and workflow processes are also imperative, given the updates and current trends in intensive care following the pandemic. Factors to consider and tackle when designing an ICU may involve a single-room design, patient-to-patient distance, and patient flow. Workflow modifications, including segregating healthcare staff and equipment based on patient colonization status or implementing “clean” and “dirty” pathways within the department, can also help reduce cross-contamination risks. Ultimately, the synergy between tailored prevention strategies and optimized ICU design holds the key to combating MDR colonization.

Mietchen et al. examined the impact of different population interaction structures on the colonization dynamics of MRSA in an ICU using stochastic compartmental modeling, a mathematical framework used to study the spread and dynamics of infectious diseases within a population [59]. While the study’s premise may appear straightforward or pathogen-specific, its potential significance cannot be understated. Specifically, this study demonstrates that the choice of population interaction structure in modeling HAIs can significantly impact the estimated effectiveness of interventions. These findings may critically impact infection control procedures and enhance the efficacy of intervention measures in contemporary ICUs. As research continues to emphasize the differential effectiveness of prevention strategies against MDR cross-contamination, the need for a comprehensive and accurate study design to precisely pinpoint the most efficient means of preventing the acquisition and spread of MDR colonization has become evident. In this same study, three models were compared: a single-staff-type model with random mixing between healthcare workers and patients, a nurse model (Nurse-MD) that separated nurses and physicians but still assumed random mixing, and a metapopulation model in which each nurse was assigned a specific group of patients [59]. The models were also sensitive to changes in parameters related to contact rates, the probability of patient colonization, and hand decontamination. The study found that the more structured models (Nurse-MD and metapopulation) resulted in fewer MRSA acquisitions than the single-staff-type model. The Nurse-MD model reduced acquisitions by 20.6%, whereas the metapopulation model reduced acquisitions by 51.4%. These data highlight the importance of accurately modeling different classes of healthcare workers, with nurse-specific parameters having a larger impact on model outcomes. Additionally, the study explored the relationship between nurse-patient interactions in the metapopulation model and found that the impact on MRSA acquisition was non-linear, with higher values of nurse–patient interaction resulting in reduced acquisition rates. Overall, the study emphasized the need for context-dependent model structures and parameterization in healthcare-associated infection modeling and concluded that simplified models assuming random mixing may overestimate infection rates and the impact of interventions, whereas more complex models that represent population mixing with higher granularity may be more representative of current care and workflow practices [59].

By considering more realistic and granular models that capture the heterogeneity of ICU patient–provider interactions, hospitals can make better-informed decisions about infection control strategies and allocate resources effectively to reduce cross-contamination. For example, if a hospital is considering implementing a program to improve nurse–patient assignment and reduce random mixing, interventions targeting the segregation of nurses and physicians may, in fact, be appropriate in terms of reducing cross-contamination, and the Nurse-MD model provides an estimate of the potential reduction in MRSA acquisitions (20.6%) [59]. This information can be used to assess the cost-effectiveness of the intervention and inform decision-making.

## 7. Antimicrobial Surveillance and Stewardship

Antimicrobial surveillance targeting ICUs is fundamental for combating MDR bacterial proliferation and spread [60]. Efficient collaboration between ICU physicians, pharmacists, microbiologists, and infection control specialists plays a pivotal role in the success of these programs [61,62]. By understanding the role, pathways, and patterns of environmental contamination in the transmission of MDR bacteria, clinicians and researchers can develop and adopt better practices to minimize the risks in ICU settings. Environmental cultures, such as swab tests, agar slides, and air and water sampling, provide valuable information on the presence and persistence of MDRs in the environment and on surfaces, thereby helping to establish a more definitive link between environmental contamination and the acquisition of these pathogens. Furthermore, frequent culturing can help assess the effectiveness of various cleaning and disinfection methods, which is essential for developing data-driven strategies to prevent transmission in ICU settings. Understanding the transmission dynamics of MDRs in ICU settings and monitoring their trends in the form of objective monitoring of cleaning procedures has reportedly contributed to significantly reducing patient-zone contamination [63]. Other objective monitoring methods of environmental hygiene include direct observation, which has been previously discussed, fluorescent markers, and adenosine triphosphate (ATP) bioluminescence [64].

Educating healthcare workers on antibiotic stewardship principles further enhances the effectiveness of preventive strategies [65]. This educational focus allows clinicians to adopt a more judicious approach toward prescribing antibiotics, an approach that is critical in minimizing the emergence, colonization, and transmission of MDR bacteria. ASPs are multifaceted strategies aimed at optimizing the use of antibiotics [66]. This can be achieved by ensuring the correct dosing, choosing the appropriate antibiotic, and regulating the duration and route of administration [67]. These elements of ASPs can collectively reduce the overall exposure to antibiotics and therefore minimize the emergence of the resistance and colonization of MDR bacteria [68]. This allows healthcare providers to identify patterns in antibiotic use and hence identify overuse or misuse. Regular feedback and prospective auditing from this surveillance can then guide alterations in prescribing habits, leading to more efficient use of antibiotics and a reduction in resistance [69].

The implementation of ASPs has been shown to have positive effects on the management of infections in ICUs. A recent study conducted across seven Italian ICUs demonstrated that a multifaceted ASP led to improvements in the management of infections, including a reduction in the duration of empirical therapy and the use of quinolones. Additionally, the proportion of MDR bacteria in ICU-acquired infections decreased, indicating a positive impact on reducing resistance [70].

However, implementing such a program can pose several challenges. For instance, it requires a significant amount of time and effort to collect, analyze, and interpret data, which may strain already limited resources. Additionally, changing long-standing prescribing habits may face resistance from some clinicians [71]. Also, the success of these programs greatly depends on close and continuous collaboration between different professionals, a challenge that may prove difficult to overcome in the hectic ICU environment.

In terms of future directions, technological advancements could play a vital role in ASPs. For example, artificial intelligence (AI) and machine learning algorithms could be used to analyze large amounts of data more efficiently, predict outbreaks, or even suggest the most appropriate antibiotics based on specific patient characteristics and local epidemiology, as evidenced by successful previous attempts in using computerized methods of surveillance [72,73,74]. Moreover, fostering a culture of perpetual stewardship among healthcare professionals based on continuous education and training can significantly improve adherence to the guidelines [75]. Multidisciplinary meetings have also been proven to be effective in reducing antibiotic use in the ICU setting [76].

Finally, while these strategies aim to combat the spread and development of MDR bacteria, specifically in the ICU setting, it is crucial to recognize that these efforts are part of a broader, ongoing commitment to global health. The importance of a One Health approach, which recognizes the interconnectedness of human, animal, and environmental health, cannot be understated in this context [77].

## 8. Systematic Approach and Integration

It becomes evident that reducing the risk of MDR bacterial transmission from personnel to patients goes beyond the standardization of cleaning practices and technological innovation, as human factors have to be taken into consideration as well. Human factor interventions, such as educational training, monitoring, and feedback, play a crucial role in reducing MDR cross-contamination [78]. Audit and feedback programs, as well as a culture of safety initiatives, have shown promising results in reducing MDR infections. Cross-contamination in the ICU is a multifactorial issue, with factors such as prior room occupants, staff members, medical procedures, and patient conditions all playing a role in the transmission of MDR pathogens. This multifactorial nature implies that prevention strategies should not solely focus on improving cleaning techniques but should also consider a more comprehensive approach to infection control. Such an approach may include optimizing staff compliance with hygiene protocols, by enhancing monitoring systems and intensifying educational efforts, and implementing targeted interventions to track and respond to potential outbreaks. By addressing the multiple factors contributing to cross-contamination, a more effective and holistic strategy can be developed to minimize the transmission of MDR pathogens in ICU settings and ultimately improve patient outcomes.

A recent systematic review has shed light on the efficacy of interventions to improve healthcare environmental hygiene (HEH) and its impact on patient colonization and HAIs [47]. This review included 26 studies that assessed various parameters, including mechanical, chemical, and human factor approaches. Overall, 58% of the studies demonstrated a significant decrease in HAI or colonization by at least one tested microorganism. Human factor interventions showed the highest proportion of significant reductions (100%), followed by chemical (86%) and mechanical (63%) interventions [47]. However, there were several limitations to this study. First, high-quality studies in the field of HEH are lacking, with most studies being retrospective or prospective before-and-after studies with limited methodological quality. Of note, only 42% of the included studies were classified as high-quality. Second, the interventions included in the studies were diverse, and the study design was largely heterogeneous because the studies used different outcome measures, such as patient colonization, healthcare-associated infections (HAI), or environmental bioburden, making it difficult to compare the results and draw consistent conclusions. Furthermore, the focus of these studies was often limited to specific microorganisms, such as MRSA and *C. difficile* and VREs. For instance, all human factor interventions included concerned *C. difficile* tackling measures and are therefore not representative of all pathogens. Therefore, the authors suggested that a combination of different interventions may be more effective in improving HEH and reducing patient colonization or HAI [47].

Another systematic review by Teerawattanapong et al. offered valuable insights into the prevention and control of MDR Gram-negative bacteria (GNB) in adult ICUs and evaluated the relative efficacy of different strategies, including standard care, ASPs, environmental cleaning, decolonization methods, and source control to prevent MDR-GNB in ICUs [79]. The findings revealed that a four-component strategy, incorporating standard care, ASPs, environmental cleaning, and source control, was the most effective intervention in preventing MDR-GNB acquisition compared with standard care alone. Notably, when environmental cleaning was added to standard care plus ASP or when source control was added to standard care plus environmental cleaning, a significant reduction in the acquisition of MDR *A. baumannii* was observed. Furthermore, strategies incorporating ASPs as a core component resulted in a significant decrease in the acquisition of extended-spectrum β-lactamase (ESBL)-producing *Enterobacteriaceae*, underlining the need to adopt a broader approach to prevent the spread of MDR bacteria in the ICU setting [79].

A study that emphasized these points was recently conducted in the neonatal intensive care unit (NICU) of “Civico” hospital in Palermo, Italy, spanning three years and enrolling 419 patients in the surveillance in order to investigate the effectiveness of a coordinated intervention strategy in reducing the prevalence of MDR-GNB carriage [80]. The intervention measures included intensified sample collection, weekly stakeholder meetings, and the improvement of prevention measures, such as antimicrobial therapy protocols, hand-washing sensitization posters, the replacement of contaminated devices, and the introduction of bundles for common procedures. A significant reduction in the prevalence of MDR-GNB, ESBL-producing GNB, and ESBL-*K. pneumoniae* carriage was observed between the pre- and post-intervention periods (*p* < 0.001). In the first year after the intervention, the prevalence of MDR-GNB and ESBL-*K. pneumoniae* carriage dropped to 20.6% and 11.1%, respectively, compared with 62.2% and 57.8%, respectively, in the pre-intervention period [80]. The second year of the post-intervention period maintained a low prevalence of MDR-GNB carriage (25.9%), with a significantly lower prevalence of ESBL-KP (3.5%) than the first post-intervention year (*p* = 0.006) [80]. These results demonstrate the long-lasting impact of a coordinated intervention strategy involving active cooperation between epidemiologists and clinicians in reducing the circulation of MDR-GNB in the NICU setting.

## 9. Policy-Making Manual

### 9.1. Framework for Developing Policy Recommendations on Preventing MDR Bacterial Transmission in ICUs

**Figure 1:**
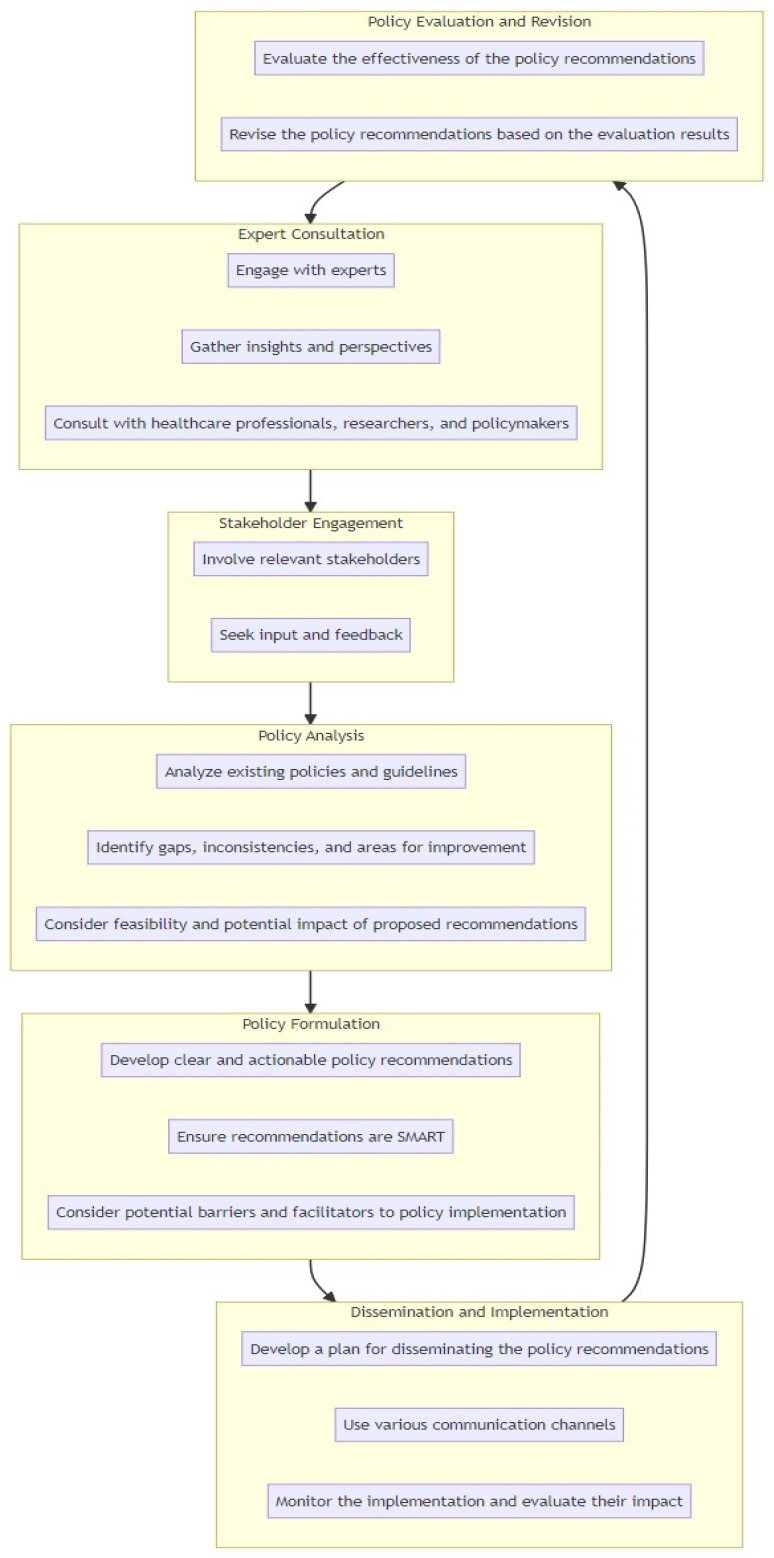
Strategic progression for formulating and implementing policies on MDR bacteria prevention in the ICU.

In Table 3 and Figure 1, we outline a systematic approach to developing policy recommendations aimed at preventing the transmission of MDR bacteria in the ICU. This approach was designed to ensure that the recommendations were evidence-based, practical, and aligned with the needs and realities of the ICU setting. The process involves several key steps including expert consultation, stakeholder engagement, policy analysis, policy formulation, dissemination and implementation, and policy evaluation and revision.

### 9.2. Proposed Policy Recommendations for Preventing MDR Bacterial Transmission in ICUs

Given the vast heterogeneity of possible MDR contraction methods in crowded and complex environments, such as ICUs, as well as the concurrent coexistence of patients with varying medical conditions and vulnerabilities, preventing MDR bacterial transmission is by definition a challenging task. Consequently, the implementation of broader and more robust infection control measures must cover multiple aspects and should not be limited to specific interventions or quantified in the form of a set of criteria. These measures include establishing comprehensive infection control guidelines, continuously monitoring and evaluating infection control practices, and fostering interdisciplinary collaboration in ICU settings. The continuous monitoring and evaluation of infection control practices are crucial for immediate identification, rectification, and learning from deviations in these practices to continually improve the healthcare environment. Fostering interdisciplinary collaboration between medical professionals provides opportunities to develop and share best practices and novel successful interventions. Emphasizing universally applicable prevention measures is highly valuable because of the diverse possible avenues of MDR bacterial transmission in ICU environments. Investing efforts in a holistic, collaborative approach to infection control is warranted, encompassing learning while tackling heterogeneity to achieve the best possible patient outcomes and safety.

By adopting a multifaceted approach that combines targeted interventions with broader preventive strategies, healthcare facilities can create a more coherent line of defense against the spread of MDR pathogens. Such a holistic approach would not only address specific transmission routes but also help build a culture of safety and accountability among healthcare personnel, ultimately leading to improved patient outcomes and a reduced burden of healthcare-associated infections.

**Figure 2:**
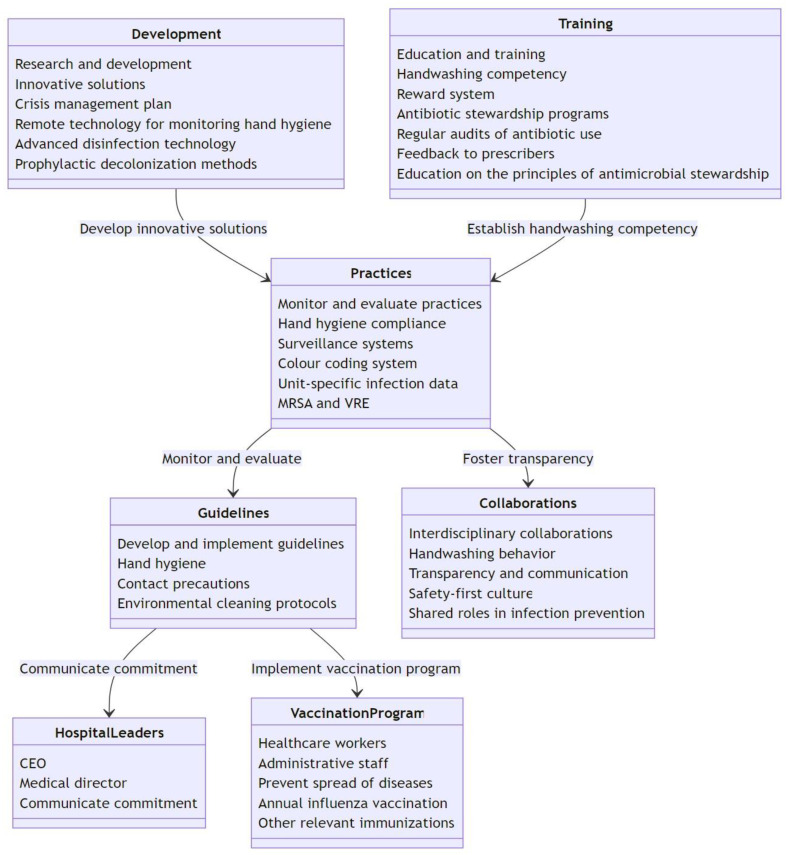
Decoding the multifaceted approach: a blueprint to combat MDR bacteria in the ICU.

Based on the collected evidence and the exploration of new technologies and strategies, we propose specific policy recommendations to guide discussions and inform decision-making processes (Figure 2). These recommendations are grounded in evidence-based practices and designed to foster a culture of safety, accountability, and continuous improvement in healthcare settings:

1. Develop and implement evidence-based infection prevention and control guidelines tailored to ICUs, including those for hand hygiene, contact precautions, and environmental cleaning protocols. Ensure that hospital leaders such as the CEO and medical director actively communicate their commitment to these guidelines and infection control measures through regular communication channels. Implement a comprehensive vaccination program for healthcare workers and administrative staff to prevent the spread of vaccine-preventable diseases in ICUs. This should include annual influenza vaccination and other relevant immunizations.
2. Continuously monitor and evaluate infection control practices, identifying areas for improvement to ensure best practices are consistently in use. Implement a system for monitoring hand hygiene compliance and provide regular feedback to clinical units, including unit-specific infection data for MRSA and VRE. Encourage the use of surveillance systems to track and monitor the prevalence and spread of multidrug-resistant organisms (MDROs) in ICUs. These data can be used to inform infection control strategies and to assess the effectiveness of interventions. Implement a color-coding system to separate different areas and prevent cross-contamination. This will make it easier for the staff to identify and adhere to specific cleaning protocols for each area. Adopt quality and cost-based selection criteria for interventions, such as annual cost reduction based on patient-day and infection reductions, use manpower, and benchmark the implementation duration for each intervention.
3. Promote strong interdisciplinary collaborations between all relevant specialists to foster a safety-first culture and shared stress roles in infection prevention in medical settings. Encourage all personnel in supervisory roles to model appropriate handwashing behavior and address poor handwashing practices among their staff, creating a culture of accountability and emphasizing the importance of hand hygiene in preventing MDR transmission. Foster a culture of transparency and open communication regarding infection control issues. This includes reporting and discussing infection rates, outbreaks, and control measures in a nonpunitive manner, which can help identify problems and solutions more effectively.
4. Allocate resources for the research and development of novel technologies and practices, targeting the reduction in environmental contamination and cross-transmission. Invest in the development and implementation of innovative solutions, such as remote technology for monitoring hand hygiene compliance, advanced disinfection technology for decontaminating surfaces, and prophylactic decolonization methods for healthcare workers, such as adequate and updated personal protective equipment and decontamination devices, such as mobile phone UV tanks. Develop and implement a robust crisis management plan to ensure the continuity of infection control measures during emergencies or outbreaks. This should include contingency plans for staff shortages, supply chain disruptions, and increased patient volume.
5. Chronically reinforce education and training in core infection prevention and control practices in sequential sessions given to ICU personnel, with a focus on their roles in enhancing both visible engagement and ensuring foundational knowledge of preventive approaches. Establish handwashing as a core competency for all clinical staff with regular assessments and the reinforcement of proper techniques. Recognize and reward individuals or teams that demonstrate exemplary hand hygiene practices through formal and informal recognition systems. Advocate for the implementation of ASPs to optimize the use of antimicrobials, reduce the risk of antibiotic resistance, and improve patient outcomes. This should include regular audits on antibiotic use, feedback to prescribers, and education on the principles of antimicrobial stewardship.

### 9.3. Proposed Program Analysis for Enforcing Hand Hygiene Standards in ICUs

The WHO’s multimodal hand hygiene improvement strategy consists of five components. The first is a system change, which involves ensuring that the necessary infrastructure for hand hygiene is in place. The second component is the training and education of healthcare workers regarding the importance of hand hygiene. The third component is the evaluation of and feedback on hand hygiene practices, infrastructure, and perceptions. The fourth component involves placing reminders in the workplace to encourage and remind healthcare workers to practice good hand hygiene. The final component is creating an institutional safety climate, which involves fostering a culture of hand hygiene and patient safety within a healthcare facility. A pilot survey based on the hand hygiene multimodal improvement strategy aimed to evaluate the state of HEH worldwide, incorporating input from 743 healthcare facilities from all World Bank income levels [81]. The results showed that almost all facilities (98%) lacked some or all of the five components of the strategy, regardless of income level [81].

Considering the aforementioned points and in an effort to emphasize the importance of evidence-based guidelines, comprehensive audit methods, the clear communication of results, the involvement of multidisciplinary specialists, and the exploration of novel technologies to improve HEH and prevent HAIs, we hereby propose a detailed program analysis designed to enforce stringent hand hygiene standards and facilitate the successful execution of targeted interventions to reduce cross-contamination:

(1) Leadership commitment: Implement a policy that requires hospital leaders, such as the CEO and medical director, to actively communicate their commitment to hand hygiene and infection control measures. This can be achieved through regular communication channels such as hospital publications, emails, and meetings. Policy discussions should explore the potential benefits and challenges of incorporating innovative technologies, such as UV or gaseous decontamination, into healthcare cleaning practices. Encourage all personnel in supervisory roles to model appropriate handwashing behaviors and address poor handwashing practices among staff. This can help to create a culture of accountability and emphasize the importance of hand hygiene in preventing MDROs transmission.
(2) Hand hygiene education: Develop a comprehensive hand hygiene education program for all ICU employees and physicians. This program should include information on the correct handwashing procedure, the importance of hand hygiene in preventing MDROs transmission, and the use of appropriate hand hygiene products. All recommendations and educational materials should be supported by evidence in the scientific literature and involve collaboration between professional societies, public institutions, and industry stakeholders. The importance of incorporating evidence-based recommendations and involving relevant specialists in educational practices cannot be overemphasized in the policymaking process.
(3) Hand hygiene competency: Establish handwashing as a core competency for all clinical staff with regular assessments and the reinforcement of proper techniques. New hires should receive handwashing fact sheets and sample hand hygiene products as part of their orientation. Training programs, educational content, and goals should be explained clearly and extensively to employees regardless of their background, stature, or hierarchical position. Ensure that the hiring and dismissal processes consider the candidate’s commitment to hand hygiene and infection control practices, reinforcing the organization’s values and expectations regarding hand hygiene and the prevention of MDROs transmission.
(4) Monitoring and feedback: Implement a system for monitoring hand hygiene compliance and providing regular feedback to clinical units. This could include unit-specific infection data for MRSA and VRE as well as using outbreaks or high infection rates as opportunities to review and reinforce handwashing practices. Different approaches should be considered to communicate feedback and possible audit results, given their potential impact on healthcare personnel and patient safety based on cultural differences, system hierarchies, and the goals of each intervention. Develop a policy to recognize and reward individuals or teams who demonstrate exemplary hand hygiene practices. This could include formal recognition such as awards or certificates or informal recognition such as praise or positive feedback from supervisors. For instance, National Health Service cleaning standards use a star rating system displayed in each area, whereas the DIN 13063 guidelines emphasize the need for clear communication of results to employees at the execution level [82].

## 10. Materials and Methods

### 10.1. Study Design and Data Collection

A comprehensive literature search was conducted by four independent reviewers using the PubMed, Scopus, and Web of Science databases to identify relevant studies investigating the role of ICU personnel in the transmission of MDR bacteria and strategies for prevention. Keywords such as “intensive care unit”, “healthcare personnel”, “multidrug-resistant bacteria”, “colonization”, and “transmission” were used. Articles published within the last few years were prioritized with a focus on systematic reviews, meta-analyses, and high-quality cohort studies. Additionally, the reference lists of the included articles were scrutinized to identify additional pertinent studies.

### 10.2. Data Analysis and Synthesis

The quality assessment and data synthesis were based on the authors’ discretion and expertise in the field, and eligibility was determined based on the relevance of the evidence to the ICU context, encompassing not only ICU-specific findings but also evidence that could potentially apply to the ICU setting. The extracted information was critically evaluated and synthesized to create a coherent narrative of the findings, incorporating the role of ICU personnel in cross-contamination, existing preventative measures, novel technologies, and strategies employed along with AMSS input, to construct effective and thoroughly described policy recommendations. The synthesis of evaluated data aims to provide a comprehensive assessment of the current knowledge on the topic, accurately identify potential gaps and/or overlooks in existing directives, and thus provide a basis for future policymaking directions.

Following data collection, large language models were used to synthesize, analyze, and format the data. The research team was responsible for verifying, validating, and interpreting data. The authors have taken full responsibility for the originality, validity, and integrity of the content of this manuscript and have assumed accountability.

### 10.3. Limitations and Gaps in the Literature

This review is based on the available literature, which may have inherent biases and limitations, including potential gaps in the research. Notably, the development of solid prediction models for patient colonization and/or infection is under-researched and requires more focused attention. We have identified two studies that provide examples of potential future directions in this research area. Çaǧlayan et al. proposed a data-driven modeling framework that can be used as a clinical decision support tool for predicting MDROs colonization and could help guide infection control measures in ICUs [83], while Wang et al. outlined a study protocol for a systematic review that aimed to summarize and assess existing models predicting MDROs colonization or infection in critically ill patients [84].

Another important gap in the literature concerns the varied impact of MDR isolates on patient mortality and potential complications. The diversity and variability of the bacteria in question necessitate further, more comprehensive research, as the data available so far have presented conflicting results [85]. While some studies suggest a direct correlation between MDR bacterial infections and increased mortality rates [86], others indicate a less clear-cut relationship [87]. Having definitive answers to this issue may help prioritize targeted interventions to reduce the spread of specific highly dangerous pathogens.

The generalizability of the suggestions presented in this review may be limited to the specific context of ICU settings. Additionally, the effectiveness of the proposed policy recommendations may vary depending on the resources and infrastructure available in different healthcare facilities. Despite these limitations, this review underscores the complexity of the issue at hand and emphasizes the need for comprehensive, tailored, and resource-sensitive solutions to effectively tackle it.

## 11. Conclusions

In the intricate ecosystem of an ICU, healthcare workers serve as inadvertent vectors for the transmission of MDR bacteria, potentially leading to HAIs and complications in critically ill patients. To effectively combat the spread of MDR bacteria and safeguard patient well-being, it is imperative to implement multilevel infection control measures. These measures encompass more than just proper hand hygiene and thorough environmental cleaning, as daily challenges in translating these best practices into consistent everyday actions among ICU personnel persist. Emerging technologies and innovations hold promise for addressing some of these issues, and their incorporation in ICUs should be investigated further. The future perspective of this study lies in the potential impact of implementing the proposed policy recommendations. By adopting a multifaceted approach that combines targeted interventions with broader preventive strategies, healthcare facilities can create a more coherent line of defense against the spread of MDROs in the ICU setting. Policies should focus on the adoption of comprehensive prevention strategies, including AMSS programs, continuous educational prompting and monitoring, and multidisciplinary initiatives, all of which are essential for optimizing counter-contamination strategies and supervising their execution in a systematic effort to improve patient outcomes in the ICU.

## Figures and Tables

**Table 1:** General recommendations for environmental cleaning and disinfection practices in the ICU [45].

1. Disinfect frequently touched surfaces or surfaces close to patients (basic hygiene), such as patient rooms, doctor’s offices, and rescue vehicles. Ensure that the disinfectant used is bactericidal and yeasticidal and allows for an appropriate exposure time before use. Clean these surfaces daily or upon patient changes (without previous isolation).
2. For infrequently touched surfaces or surfaces distant from patients, such as clean work surfaces, medication tables, and dressing trolleys, ensure that the disinfectant used is bactericidal and yeasticidal. Clean these surfaces only when necessary, such as when they are visibly dirty, immediately before use, or in situations of increased risk of contamination. Base the exposure time on the allowed exposure time for a specific disinfectant.
3. Disinfect floor surfaces daily to ensure a clean and hygienic environment, thus minimizing the risk of infection spreading within the ICU.
4. Pay attention to clean work areas/rooms and ensure the proper disinfection of surfaces before performing aseptic tasks.
5. Compliance with other area-specific cleaning regulations (e.g., blood banks, hospital pharmacies, doctor’s offices) is critical for maintaining a safe environment in the ICU.
6. After the discharge of a patient from the ICU, perform a final disinfection of all surfaces to prepare for the next patient and reduce the risk of cross-contamination.

**Table 2:** Recommendations for environmental cleaning and disinfection practices in high-risk hospital areas in a developing country [46].

1. Frequency of cleaning: clean high-risk areas such as ICUs once every two hours, with spot cleaning as required.
2. Level of cleaning/disinfection: perform both cleaning and intermediate-level disinfection in high-risk areas. Soap and detergent are used for cleaning, followed by disinfection with alcohol and aldehydes.
3. Method of cleaning/disinfection: use a combination of soap and detergent for cleaning surfaces, followed by the application of disinfectants containing alcohol and aldehyde compounds to achieve intermediate-level disinfection.
4. Evaluation/auditing frequency: conduct weekly evaluations and audits of the cleaning and disinfection practices in high-risk areas. This should be done by the officer in charge of the Sanitation and Infection Control Team.
5. Staffing: in the ICU, one Sanitary Attendant is allocated up to six ICU beds in each shift.
6. Induction training: provide 24 h of intensive training on general cleaning and infection control to all cleaning staff involved in high-risk areas. This training should be followed by seven days of supervised duties.
7. Refresher training/on-the-job training: conduct 4 h of training every month to refresh the knowledge and skills of the cleaning staff in high-risk areas.

**Table 3:** Framework for developing policy recommendations on preventing MDR bacterial transmission in the ICU.

*Expert Consultation for Policy Development*: Engage with experts in the field of infection control, healthcare policy, and ICU management to gather insights and perspectives on the topic. Consultations with healthcare professionals, researchers, and policymakers can provide valuable input for developing policy recommendations.
*Stakeholder Engagement in Policy Formulation*: Involve relevant stakeholders, including healthcare administrators, infection control specialists, ICU staff, and patient representatives in the policy development process. Seek their input and feedback to ensure that the recommendations are practical, feasible, and aligned with the needs and realities of the ICU.
*Policy Analysis: Identifying Gaps and Opportunities*: Analyze existing policies and guidelines related to infection control in ICUs at local, national, and international levels. Identify gaps, inconsistencies, and areas for improvement in current policies. Consider the feasibility and potential impact of proposed policy recommendations in the context of existing regulations and healthcare systems.
*Formulating SMART Policy Recommendations*: Based on the evidence from the literature review, expert consultation, stakeholder engagement, and policy analysis, develop clear and actionable policy recommendations. Ensure that the recommendations are specific, measurable, achievable, relevant, and time-bound (SMART). Consider the potential barriers and facilitators of policy implementation and develop strategies to address them.
*Dissemination and Implementation of Policy Recommendations*: Develop a plan for disseminating policy recommendations to relevant stakeholders, including healthcare facilities, professional organizations, and government agencies. Consider the use of various communication channels such as policy briefs, guidelines, workshops, and conferences to raise awareness and promote the adoption of recommendations. Monitor the implementation of the policy recommendations and evaluate their impact on MDR bacterial transmission in the ICU.
*Policy Evaluation and Revision: A continuous process*: Regularly evaluate the effectiveness of policy recommendations in reducing multidrug-resistant bacterial transmission in the ICU. Collect data on key indicators, such as infection rates, hand hygiene compliance, and environmental cleanliness, to assess the impact of the policy. Revise and update policy recommendations based on new evidence, emerging technologies, and changing healthcare practices.

## Data Availability

Not applicable.

## Abbreviations

ABHS	Alcohol-Based Hand Sanitizers
AHHMS	Automated Hand Hygiene Monitoring System
AI	Artificial Intelligence
AMSS	Antimicrobial Surveillance and Stewardship
ASP	Antimicrobial Stewardship Program
ATP	Adenosine Triphosphate
ATS	American Thoracic Society
CDC	Centers for Disease Control and Prevention
EHHMS	Electronic Hand Hygiene Monitoring System
ESBL	Extended-Spectrum β-Lactamase
GNB	Gram-Negative Bacteria
HAC	Healthcare-Acquired Colonization
HAI	Healthcare-Associated Infection
HCP	Healthcare Personnel
HEH	Healthcare Environmental Hygiene
HEPA	High-Efficiency Particulate Absorption
ICU	Intensive Care Unit
IDSA	Infectious Diseases Society of America
MD	Model
MDR	Multidrug-Resistant
MDRO	Multidrug-Resistant Organism
MRSA	Methicillin-Resistant *Staphylococcus aureus*
NICU	Neonatal Intensive Care Unit
PPE	Personal Protective Equipment
SHEA	Society for Healthcare Epidemiology of America
UV	Ultraviolet
VRE	Vancomycin-Resistant enterococci
WHO	World Health Organization
